# Two Infants with Presumed Congenital Zika Syndrome, Brownsville, Texas, USA, 2016–2017

**DOI:** 10.3201/eid2404.171545

**Published:** 2018-04

**Authors:** Ashley Howard, John Visintine, Jaime Fergie, Miguel Deleon

**Affiliations:** Driscoll Children’s Hospital, Corpus Christi, Texas, USA

**Keywords:** Zika virus, viruses, flavivirus, infants, newborns, pregnancy, congenital Zika syndrome, microcephaly, southern Texas, Brownsville, Texas, United States, Mexico

## Abstract

Since 2007, Zika virus has spread through the Pacific Islands and the Americas. Beginning in 2016, women in Brownsville, Texas, USA, were identified as possibly being exposed to Zika virus during pregnancy. We identified 18 pregnant women during 2016–2017 who had supportive serologic or molecular test results indicating Zika virus or flavivirus infection. Two infants were evaluated for congenital Zika syndrome after identification of prenatal microcephaly. Despite standard of care testing of mothers and neonates, comparative results were unreliable for mothers and infants, which highlights the need for clinical and epidemiologic evidence for an accurate diagnosis. A high index of suspicion for congenital Zika syndrome for at-risk populations is useful because of current limitations of testing.

Zika virus is an arbovirus and flavivirus transmitted by *Aedes aegypti* and *Ae. albopictus* mosquitoes, vectors that also transmit other arboviruses, such as dengue virus and chikungunya virus. Zika virus was discovered in the Zika Forest of Uganda in 1947 in rhesus and macaque monkey populations (http://www.who.int/emergencies/zika-virus/timeline/en/). Until 2007, only 14 cases of human infection were reported in Asia and Africa (*1*). However, outbreaks of infection with Zika virus occurred on Yap Island, Micronesia, in 2007 and in French Polynesia in 2013, affecting ≈31,000 persons (*2*). Zika has spread rapidly in the Americas since 2015 and has been associated with hundreds of confirmed microcephaly cases in Brazil, Colombia, and Puerto Rico (*2**–**7*). In April 2016, the Centers for Disease Control and Prevention (CDC) confirmed evidence that supported the causal relationship between Zika virus infection prenatally and microcephaly, in addition to other brain abnormalities, and described what has become known as congenital Zika syndrome (*2**,**8**–**11*).

In the United States since June 2017, there have been 5,335 travel-associated cases and 227 locally transmitted cases of infection with Zika virus in southern Florida and Brownsville, Texas (*4*). A total of 2,364 pregnant women (972 completed pregnancies) with laboratory evidence of Zika virus infection in the United States have been reported to CDC; the Zika-related birth defect risk among these women has been estimated to be 1 in 10 women (*12**,**13*). In November 2016, local transmission was confirmed by health authorities in Brownsville, and screening for Zika virus in asymptomatic pregnant patients and testing for Zika virus in symptomatic patients began (*14**,**15*). This screening was quickly followed in December 2016 by identification of pregnant women with supportive laboratory evidence of Zika virus infection in the Brownsville area.

## Cases

Eighteen cases of possible Zika virus infection in pregnant women were identified by screening and testing of symptomatic patients living in Brownsville during December 2016–May 2017. Twelve case-patients had laboratory evidence of Zika virus infection: positive PCR results for serum (*8*), serum and urine (*3*), or placenta (*1*). One case-patient had plaque reduction neutralization test (PRNT) results consistent with recent Zika virus infection, and 5 case-patients had PRNT results consistent with recent flavivirus infection. Fifteen women had delivered their babies as of July 14, 2017; the remaining women had estimated dates of delivery through early 2018. Two pregnant women in this cohort had findings consistent with congenital Zika syndrome. Neonatal and infant follow-up is ongoing for women who delivered up to this point. We report the prenatal and neonatal outcomes for 2 infants who had congenital Zika syndrome.

## Case-Patient 1

**Figure 1:**
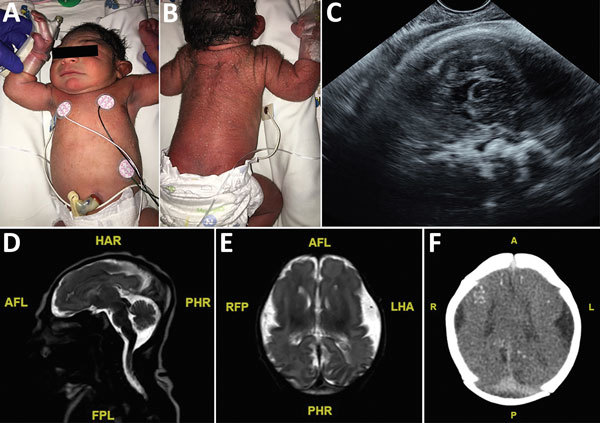
Term male infant (case-patient 1) with presumed congenital Zika syndrome, Brownsville, Texas, USA, 2016–2017. A) Microcephaly on the day of birth. Head circumference was 29 cm, which is 2.63 SDs below the mean value for term male newborns. Craniofacial abnormalities present are mild narrow and laterally depressed frontal bone and mild retrognathia. B) Generalized pustular melanosis rash. C) Prenatal transvaginal ultrasonographic (midsagittal plane) image at 37.2 weeks’ gestation, showing calcifications at the gray matter–white matter junction. Head circumference was 251 mm. D) Sagittal T2 magnetic resonance image on day of life 1, showing severe microcephaly, frontal lobe polymicrogyria, and hypoplastic corpus callosum. E) Axial T2 magnetic resonance image on day of life 1, showing severely hypoplastic cerebral hemispheres and corpus callosum. Symmetric frontal lobe polymicrogyria and simplified gyral pattern in the occipital and temporal lobes are present. F) Axial computed tomography image on day of life 3, showing small bilateral brain hemispheres and hypogyration of the cerebral cortex. Areas of punctate calcification located at the subcortical and gray matter–white matter junctions of the frontal, parietal, and occipital lobes are present. A, anterior; AFL, anterior left; FPL, posterior left; HAR, anterior right; L, left; LHA, left anterior; P, posterior; PHR, posterior right; R, right; RFP, right posterior.

Case-patient 1 was born to a 23-year-old woman (G1P1) who spent the first 4 months of her pregnancy in Matamoros, Mexico. She received prenatal Zika testing while residing there, and results were negative. She moved to Brownsville, where she received prenatal care at 28 weeks’ gestation. She was screened for Zika virus by serum IgM testing; results were negative. She was referred for maternal fetal medicine at 36 weeks’ gestation because of suspected microcephaly. The fetus was found to have microcephaly: head circumference (HC) 251 mm, which was 5 SD below the mean value. The mother denied having any symptoms of Zika virus infection (rash, fever, malaise, arthralgia, or conjunctivitis). At 37 weeks’ gestation, transvaginal fetal neuroimaging was performed; results showed calcifications in the cortical white matter–gray matter junction, but no calcifications were observed in the thalami (Figure 1, panel C). On the basis of ultrasonographic findings, a maternal repeat Zika virus IgM test was performed and showed a positive result at 37 weeks’ gestation. PRNT results were consistent with recent flavivirus infection (Zika and dengue PRNT titers >1,280) (*16*). A TORCH (toxoplasmosis, rubella cytomegalovirus, herpes simplex virus, and HIV) panel did not show evidence of recent infections, and results of a cell-free fetal DNA screening were negative.

An elective primary cesarean delivery was performed at 39 weeks’ gestation. APGAR scores for the baby were 9 at 1 min and 9 at 5 min. At initial examination, the neonate had a vesicular generalized rash, overriding sutures, and microcephaly. The initial HC of the infant was 29 cm, which was 2.63 SD below the mean value for term male newborns. Birthweight was 2.62 kg (4.76 percentile), and birth length was 45 cm (3.2 percentile). On further examination, mild craniofacial disproportion with narrow and laterally depressed frontal bone and mild retrognathia was seen. No limb contractures were observed (Figure 1, panel A).

The newborn was transferred to the neonatal intensive care unit (NICU) at Driscoll Children’s Hospital (Corpus Christi, TX, USA) on day 1 of life. Because of a generalized vesicular rash, concern for herpes simplex virus infection prompted treatment with acyclovir for the first 2 days of life. The rash was diagnosed as neonatal pustular melanosis; it faded by day 1 of life and disappeared by day 2 of life (Figure 1, panels A, B). Zika virus testing was performed on day 1 of life. Zika virus PCRs were performed for serum, urine, and cerebrospinal fluid (CSF); all results were negative. Zika virus IgM testing was ordered for serum and CSF, but the test for CSF was not performed by the state laboratory because of a negative PCR result for CSF. Serum was positive for Zika virus IgM, which is consistent with probable congenital Zika virus infection. Results of placental testing by reverse transcription PCR for the Zika virus nonstructural protein 5 gene were positive. Test results for dengue and chikungunya viruses were negative. Additional TORCH testing was performed, and results were negative for herpes simplex virus, cytomegalovirus, syphilis, HIV, *Toxoplasma* spp., and parvovirus.

The neonate passed the initial newborn hearing screen and had a pediatric ophthalmologic examination on day 1 of life, during which a small left subconjunctival hemorrhage was identified (*17**,**18*). Initial head ultrasonography on day 1 of life showed parietal calcifications and pachygyria. Follow-up magnetic resonance imaging showed frontal lobe polymicrogyria, bilateral dystrophic calcifications, and severe microcephaly (Figure 1, panels D, E). Computerized tomography was performed on day 3 of life for better characterization of calcifications and showed bilateral small brain hemispheres with hypogyration of the cerebral cortex. Areas of punctate calcification were observed at the subcortical and gray matter–white matter junctions of the frontal, parietal, and occipital lobes (Figure 1, panel F). A prominent occipital bone was observed with overlapping of the region of the lambdoid suture and prominent bony ridging at the region of the coronal sutures. Partial fusion of the inferior aspect of coronal sutures and asymmetric closure of the temporal sutures were also observed. There was no ventriculomegaly.

The infant was in the NICU for 9 days. During that time, the infant had poor feeding and required an orogastric tube to assist with feeds until day 7 of life. The CDC recommended electroencephalogram (EEG) testing because of new information concerning development of seizures in 30%–50% of infants with congenital Zika syndrome; the EEG result was unremarkable (*19**,**20*). Microarray and microcephaly gene panel were tested; all showed negative results. A screening echocardiogram showed results consistent with reference transitional neonatal cardiac changes. Results of thyroid function testing, complete blood count, and a comprehensive metabolic panel (CMP) were all within reference ranges.

The infant was discharged on day 9 of life. At discharge, he had an HC of 30 cm, which was 3.16 SD below the mean value for term male newborns with microcephaly.

## Case-Patient 2

**Figure 2:**
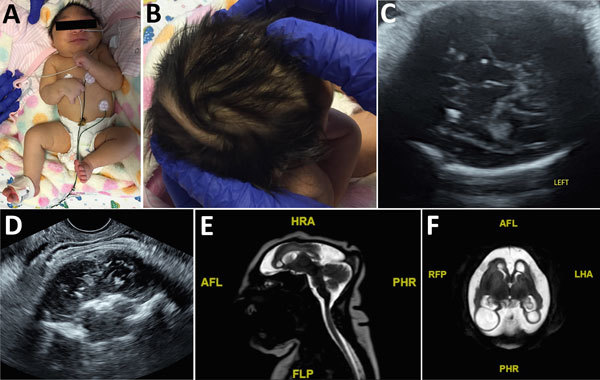
Term female infant (case-patient 2) with presumed congenital Zika syndrome, Brownsville, Texas, USA, 2016–2017. A) Microcephaly on the day of birth. Head circumference was 26.5 cm, which is 6.23 SDs below the mean value for term females. Craniofacial disproportion with narrow and laterally depressed frontal bone is seen. Upper wrist contractures are present, more apparent on the right, with ulnar deviation. B) Redundant scalp skin with multiple rugae. C) Transabdominal ultrasonography image of the axial transthalamic plane at 37 weeks’ gestation, showing coarse bilateral calcifications in the thalami. D) Transvaginal ultrasonography image of the coronal section at 37 weeks’ gestation, showing coarse calcifications in the thalami. E) Sagittal T2 turbo spin echo magnetic resonance image on day of birth, showing microcephaly, dysgenesis of the corpus callosum, and a small bilateral choroid plexus cyst. F) Axial T2 turbo spin echo magnetic resonance image on day of birth, showing microcephaly with enlarged extra-axial spaces and a smooth gyral pattern. Large bilateral posterior parietal and occipital lobe parenchymal cysts are present. AFL, anterior left; FLP, left posterior; HRA, right anterior; LHA, left anterior; PHR, posterior right; R, right; RFP, right posterior.

Case-patient 2 was born to an 18-year-old woman (G1P1) who lived in Brownsville. She reported weekly travel to Matamoros, Mexico, during the early stages of her pregnancy. She denied any viral symptoms of rash, fever, malaise, arthralgia, or conjunctivitis. She was screened by her obstetrician for Zika virus at 23 weeks’ gestation by a PCR for serum; results were positive. Results were negative for a Zika virus PCR for urine and serum Zika virus IgM. At 28 weeks’ gestation, fetal ultrasonography was performed for growth and anatomy evaluation. The fetus had microcephaly and was referred for maternal fetal medicine evaluation. The HC of the fetus was 203 mm at 29 weeks’ gestation, which was 4−5 SD below the mean value. Coarse calcifications were observed in the basal ganglia and thalami by transabdominal and transvaginal fetal neuroimaging (Figure 2, panels C, D). The TORCH panel did not show evidence of recent infections.

A planned primary cesarean delivery was performed at 39 weeks’ gestation. APGAR scores were 9 at 1 min and 9 at 5 min. At initial examination, the neonate had a prominent sagittal ridge, overriding sutures, and severe microcephaly (Figure 2, panel A). Initial head circumference was 26.5 cm, which was 6.23 SD below the mean value for term female newborns. Birthweight was 2.39 kg (2.21 percentile), and birth length was 41.5 cm (<0.01 percentile). Further examination showed excess scalp skin (Figure 2, panel B) and craniofacial disproportion with narrow and laterally depressed frontal bone (Figure 2, panel A). Upper limb contractures were also observed (Figure 2, panel A). 

The patient was transferred to the NICU at Driscoll Children’s Hospital on day 1 of life, and Zika virus testing was performed the same day. Results of Zika virus PCRs were negative for serum, urine, and CSF. IgM serum was negative for Zika virus. Testing for Zika virus IgM was ordered for serum and CSF, but the test for CSF was not performed by the state laboratory because of a negative PCR result for CSF. Test results were negative for dengue virus and chikungunya virus. Additional TORCH testing was performed, and results were negative for CMV, syphilis, HIV, *Toxoplasma* spp., and parvovirus. The infant passed the initial newborn hearing screen and had a pediatric ophthalmology examination performed on day 1 of life; no eye anomalies were identified (*17*,*18*).

Initial ultrasonography of the head on day 1 of life could not be completed because the anterior fontanelle was too small. Magnetic resonance imaging showed microcephaly with enlarged extraaxial spaces, large bilateral parenchymal cysts in the posterior parietal and occipital lobes, an overall smooth gyral pattern, dysgenesis of the corpus callosum, and 5 small bilateral choroid plexus cysts (Figure 2, panels E, F).

The infant was in the NICU for 28 days, during which daily examinations showed intermittent tremors, hypertonia, and an exaggerated Moro reflex. Upper bilateral wrists continued to be contracted in the flexed and ulnar deviated positions and required physical therapy intervention. The infant had to be fed by an orogastric tube because of poor feeding until she was able to be transitioned to ad libitum feeds on day 25 of life. Because of excessive irritability and crying, the infant was given phenobarbital on day 16 of life. In addition, an EEG was performed because of tremor activity; results were uneventful. Screening echocardiogram results were consistent with standard transitional neonatal cardiac changes. Abdominal ultrasonography was performed and results were unremarkable. Results were negative for a microarray and microcephaly gene panel testing. Results of thyroid function testing, complete blood count, and a comprehensive metabolic panel were all within reference ranges.

The infant was discharged on day 27 of life. She had an HC of 27 cm, which was 7.42 SD below the mean value for term females.

## Discussion

Making a diagnosis of congenital Zika syndrome is challenging, despite testing and imaging available in a well-resourced area, such as the United States, which emphasizes the role of clinical and epidemiologic circumstances as critical pieces for a presumptive diagnosis. Diagnosis is needed not only epidemiologically, but also longitudinally for follow-up of associated problems with congenital Zika virus infection, which have been reported as a constellation of malformations and clinical symptoms involving the brain, craniofacial defects, nervous system, eyes, and limbs (*3**,**5**–**9**,**12**,**19**–**24*).

Both infants reported in our case series had findings of congenital Zika syndrome (Figures 1, 2). Results of neuroimaging performed prenatally for both infants were consistent with the presence and degree of microcephaly observed postnatally (*25*). Calcifications identified prenatally in case-patient 1 had consistent postnatal distribution at the subcortical white matter–gray matter junction. Case-patient 2 had changes in the presence of calcifications seen during prenatal ultrasonography that were not present by postnatal imaging. In addition, prenatal diagnosis of arthrogryposis was not made because of spontaneous movement of all extremities on prenatal ultrasonographic images. This limitation illustrates that the spectrum of congenital Zika syndrome cannot be fully assessed until further postnatal assessment and highlights the need for advanced neuroimaging.

However, despite the neonatal diagnosis of congenital Zika syndrome, results for maternal testing were not consistent. The first case-patient had maternal laboratory findings of probable flavivirus infection that was not identified until the third trimester. The first IgM screening (at 28 weeks’ gestation) might have shown a false-negative result, or the infection might have occurred later. However, even without definitive evidence of maternal Zika virus infection at the time of delivery, the neonate showed a positive result for Zika IgM in serum, and a subsequent placental test showed a positive result, which confirmed maternal infection.

Maternal diagnosis for case-patient 2 was confirmed with positive PCR results for serum at 23 weeks’ gestation. However, despite this newborn displaying more severe features of congenital Zika syndrome postnatally (redundant scalp skin, bilateral upper arm arthrogryposis, smaller head size, and extrapyramidal symptoms), results of serum testing for Zika virus infection were negative.

In conclusion, results for these 2 case-patients indicate the complexity and challenges of screening and diagnostic testing for congenital Zika syndrome and illustrate the need for clinical findings and epidemiologic history. We advise a high index of suspicion for congenital Zika syndrome for at-risk populations on the basis of current limitations of testing.

## References

[1] World Health Organization. Zika: the origin and spread of mosquito-borne virus, February 9, 2016 [cited 2016 Sep 12]. http://www.who.int/bulletin/online_first/16-171082/en/10.2471/BLT.16.171082 PMC5034643 27708473

[2] Panchaud A, Stojanov M, Ammerdorffer A, Vouga M, Baud D. Emerging role of Zika virus in adverse fetal and neonatal outcomes. Clin Microbiol Rev. 2016;29:659–94. 10.1128/CMR.00014-16 27281741 PMC4978612

[3] World Health Organization. Defining the syndrome associated with congenital Zika virus infection, June 2016 [cited 2016 Sep 12]. http://www.who.int/bulletin/volumes/94/6/16-176990/en/10.2471/BLT.16.176990 PMC4890216 27274588

[4] Centers for Disease Control and Prevention. Cumulative Zika virus disease case in the United States, 2015–2017 [cited 2017 Aug 31]. https://www.cdc.gov/zika/reporting/case-counts.html

[5] Cuevas EL, Tong VT, Rozo N, Valencia D, Pacheco O, Gilboa SM, et al. Preliminary report of microcephaly potentially associated with Zika virus infection during pregnancy—Colombia, January–November 2016. MMWR Morb Mortal Wkly Rep. 2016;65:1409–13. 10.15585/mmwr.mm6549e1 27977645

[6] Shapiro-Mendoza CK, Rice ME, Galang RR, Fulton AC, VanMaldeghem K, Prado MV, et al.; Zika Pregnancy and Infant Registries Working Group. Pregnancy outcomes after maternal Zika virus infection during pregnancy—US Territories, January 1, 2016–April 25, 2017. MMWR Morb Mortal Wkly Rep. 2017;66:615–21. 10.15585/mmwr.mm6623e1 28617773 PMC5657842

[7] Brasil P, Pereira JP Jr, Moreira ME, Ribeiro Nogueira RM, Damasceno L, Wakimoto M, et al. Zika virus infection in pregnant women in Rio de Janeiro. N Engl J Med. 2016;375:2321–34. 10.1056/NEJMoa1602412 26943629 PMC5323261

[8] Centers for Disease Control and Prevention. CDC concludes that Zika causes microcephaly and other birth defects [cited 2016 Sep 12]. http://www.cdc.gov/media/releases/2016/s0413-zika-microcephaly.html

[9] Rasmussen SA, Jamieson DJ, Honein MA, Petersen LR. Zika virus and birth defects: reviewing the evidence for causality. N Engl J Med. 2016;374:1981–7. 10.1056/NEJMsr1604338 27074377

[10] Simeone RM, Shapiro-Mendoza CK, Meaney-Delman D, Petersen EE, Galang RR, Oduyebo T, et al.; Zika and Pregnancy Working Group. Possible Zika virus infection among pregnant women—United States and territories, May 2016. MMWR Morb Mortal Wkly Rep. 2016;65:514–9. 10.15585/mmwr.mm6520e1 27248295

[11] Meaney-Delman D, Hills SL, Williams C, Galang RR, Iyengar P, Hennenfent AK, et al. Zika virus infection among US pregnant travelers—August 2015–February 2016. MMWR Morb Mortal Wkly Rep. 2016;65:211–4. 10.15585/mmwr.mm6508e1 26938703

[12] Reynolds MR, Jones AM, Petersen EE, Lee EH, Rice ME, Bingham A, et al.; U.S. Zika Pregnancy Registry Collaboration. Vital signs: update on Zika virus–associated birth defects and evaluation of all US infants with congenital Zika virus exposure—US Zika pregnancy registry, 2016. MMWR Morb Mortal Wkly Rep. 2017;66:366–73. 10.15585/mmwr.mm6613e1 28384133 PMC5657905

[13] Centers for Disease Control and Prevention. Pregnant women with any laboratory evidence of possible Zika virus infection in the United States and Territories, 2016–2017 [cited 2017 Aug 31]. https://www.cdc.gov/zika/geo/pregwomen-uscases.html

[14] Honein MA, Dawson AL, Petersen EE, Jones AM, Lee EH, Yazdy MM, et al.; US Zika Pregnancy Registry Collaboration. Birth defects among fetuses and infants of US women with evidence of possible Zika virus infection during pregnancy. JAMA. 2017;317:59–68. 10.1001/jama.2016.19006 27960197

[15] Oduyebo T, Igbinosa I, Petersen EE, Polen KN, Pillai SK, Ailes EC, et al. Update: interim guidance for health care providers caring for pregnant women with possible Zika virus exposure—United States, July 2016. MMWR Morb Mortal Wkly Rep. 2016;65:739–44. 10.15585/mmwr.mm6529e1 27467820

[16] Rabe IB, Staples JE, Villanueva J, Hummel KB, Johnson JA, Rose L, et al.; MTS. MTS. Interim guidance for interpretation of Zika virus antibody test results. MMWR Morb Mortal Wkly Rep. 2016;65:543–6. 10.15585/mmwr.mm6521e1 27254248

[17] Russell K, Oliver SE, Lewis L, Barfield WD, Cragan J, Meaney-Delman D, et al.; Contributors. Update: interim guidance for the evaluation and management of infants with possible congenital Zika virus infection—United States, August 2016. MMWR Morb Mortal Wkly Rep. 2016;65:870–8. 10.15585/mmwr.mm6533e2 27559830

[18] Ventura CV, Maia M, Travassos SB, Martins TT, Patriota F, Nunes ME, et al. Risk factors associated with the ophthalmoscopic findings identified in infants with presumed Zika virus congenital infection. JAMA Ophthalmol. 2016;134:912–8. 10.1001/jamaophthalmol.2016.1784 27228275

[19] Moura da Silva AA, Ganz JS, Sousa PD, Doriqui MJ, Ribeiro MR, Branco MD, et al. Early growth and neurologic outcomes of infants with probable congenital Zika virus syndrome. Emerg Infect Dis. 2016;22:1953–6. 10.3201/eid2211.160956 27767931 PMC5088045

[20] Alves LV, Cruz DD, Linden AM, Falbo AR, Melo MJ, Paredes CE, et al. Epileptic seizures in children with congenital Zika virus syndrome. Rev Bras Saude Mater Infant. 2016;16(Suppl 1):S27–31. 10.1590/1806-9304201600s100003

[21] Soares de Oliveira-Szejnfeld P, Levine D, Melo AS, Amorim MM, Batista AG, Chimelli L, et al. Congenital brain abnormalities and Zika virus: what the radiologist can expect to see prenatally and postnatally. Radiology. 2016;281:203–18. 10.1148/radiol.2016161584 27552432

[22] Del Campo M, Feitosa IM, Ribeiro EM, Horovitz DD, Pessoa AL, França GV, et al.; Zika Embryopathy Task Force-Brazilian Society of Medical Genetics ZETF-SBGM. The phenotypic spectrum of congenital Zika syndrome. Am J Med Genet A. 2017;173:841–57. 10.1002/ajmg.a.38170 28328129

[23] Eppes C, Rac M, Dunn J, Versalovic J, Murray KO, Suter MA, et al. Testing for Zika virus infection in pregnancy: key concepts to deal with an emerging epidemic. Am J Obstet Gynecol. 2017;216:209–25. 10.1016/j.ajog.2017.01.020 28126366

[24] Moore CA, Staples JE, Dobyns WB, Pessoa A, Ventura CV, Fonseca EB, et al. Characterizing the pattern of anomalies in congenital Zika syndrome for pediatric clinicians. JAMA Pediatr. 2017;171:288–95. 10.1001/jamapediatrics.2016.3982 27812690 PMC5561417

[25] Chervenak FA, Rosenberg J, Brightman RC, Chitkara U, Jeanty P. A prospective study of the accuracy of ultrasound in predicting fetal microcephaly. Obstet Gynecol. 1987;69:908–10. 3554067

